# Supplementary material for: Engineered Membranes for Residual Cell Trapping on Microfluidic Blood Plasma Separation Systems: A Comparison between Porous and Nanofibrous Membranes
Source: Membranes (Basel). 2021 Aug 31;11(9):680. doi: 10.3390/membranes11090680 (PMC8470088; doi:10.3390/membranes11090680)
Supplement: Supplementary file 1 [file membranes-11-00680-s001.zip › membranes-1356479-supplementary.pdf]

SUPPORTING INFORMATION

Engineered Membranes for Residual Cell Trapping on Microfluidic Blood Plasma Separation Systems. A Comparison Between Porous and Nanofibrous Membranes

Francesco Lopresti [1,*], Ieva Keraite [2], Alfredo Edoardo Ongaro [2], Nicola Marie Howarth [2], Vincenzo La Carrubba [1] and Maïwenn Kersaudy-Kerhoas [2,*]

[1] Department of Engineering, University of Palermo, RU INSTM of Palermo, Viale delle Scienze, 90128 Palermo, Italy; vincenzo.lacarrubba@unipa.it (V.L.C.)

[2] Institute of Biological Chemistry, Biophysics and Bioengineering, Heriot-Watt University, EH14 4AS, UK; ik22@hw.ac.uk (I.K.); ao37@hw.ac.uk (A.E.O.); N.M.Howarth@hw.ac.uk (N.M.H.)

* Correspondence: francesco.lopresti@unipa.it (F.L.); M.Kersaudy-Kerhoas@hw.ac.uk (M.K.-K.)

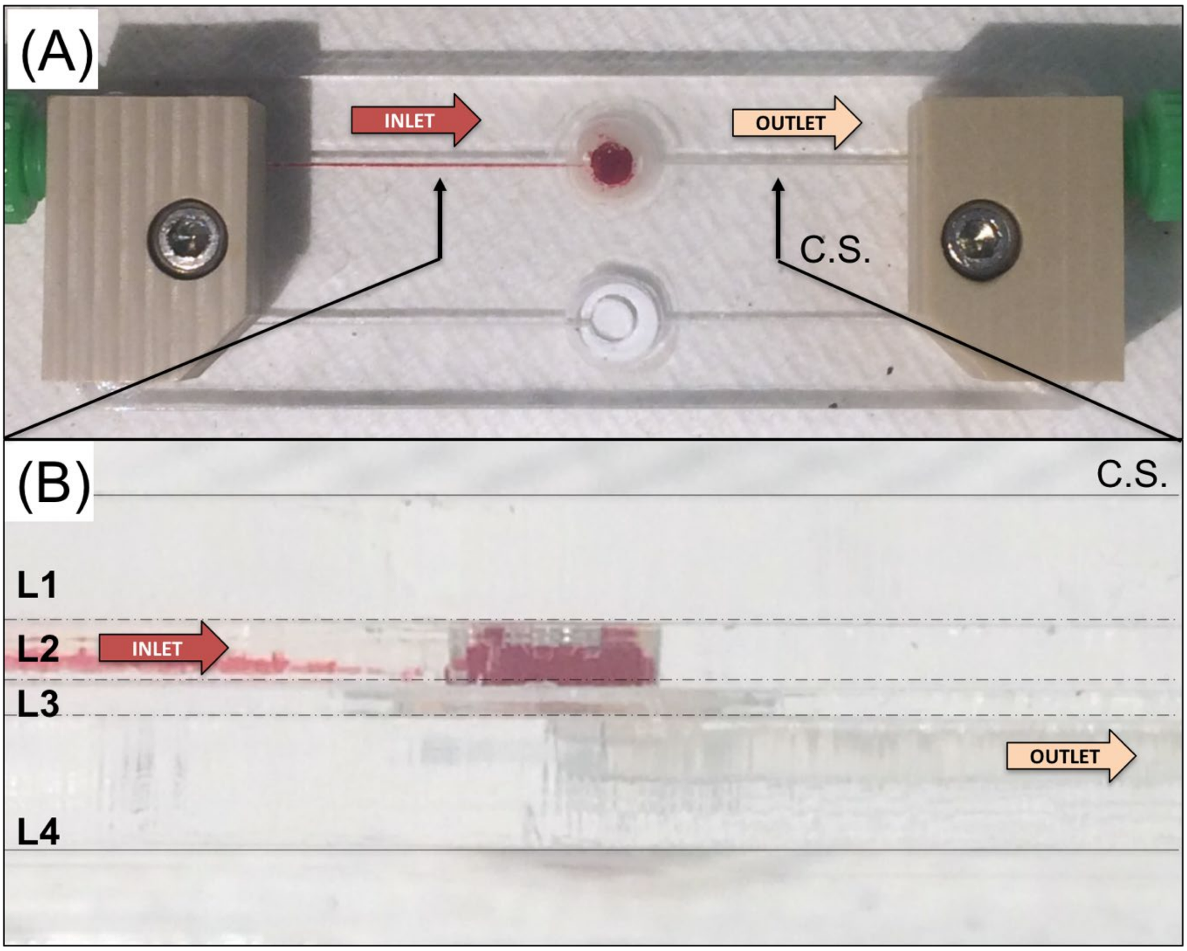

Figure S1: -Pictures of A) Top view and B) cross section of the assembled microfiltration unit.

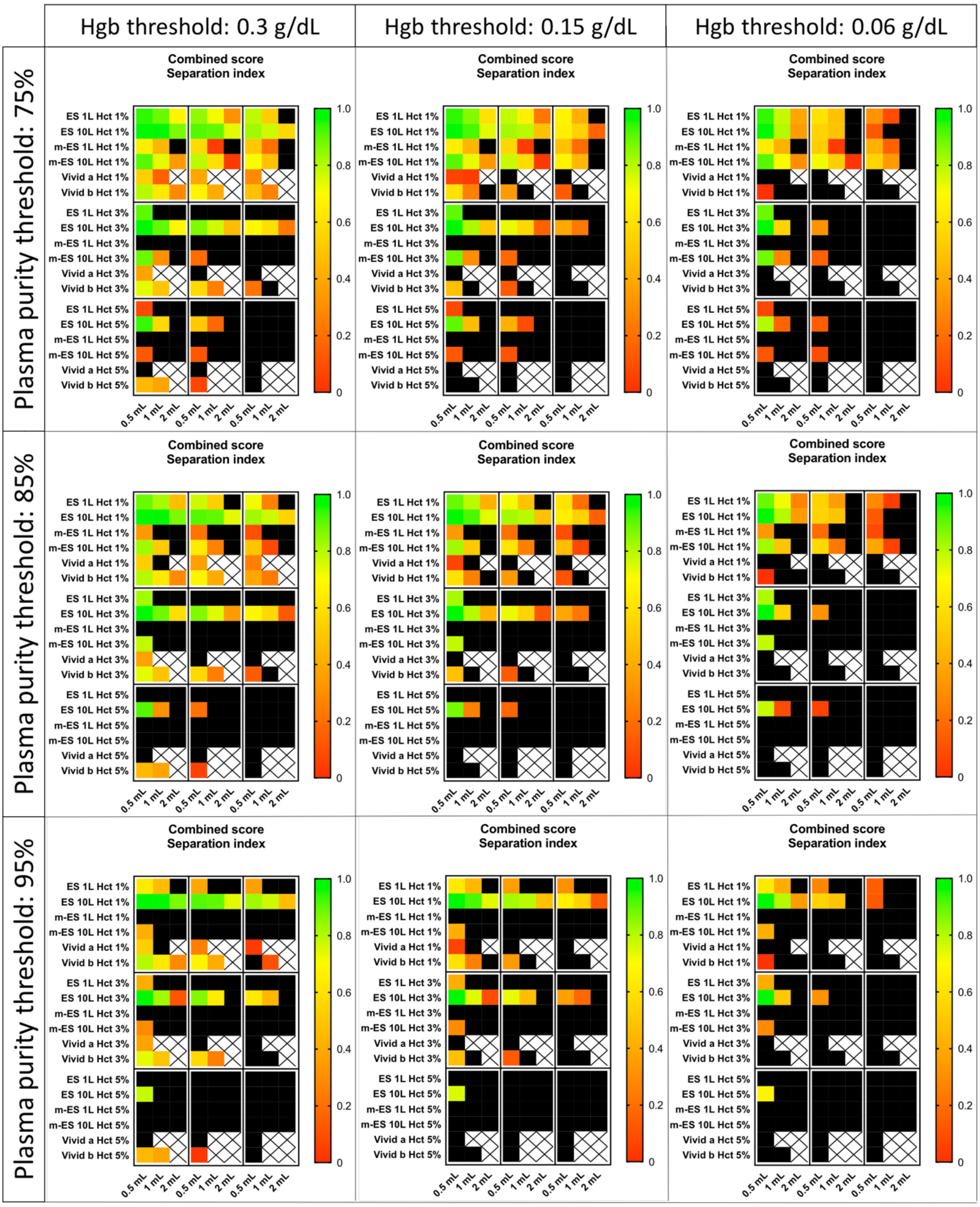

Figure S2: Combined score separation index heat maps evaluated according to Equation 8 as a function of different plasma purity and free Hgb threshold values.

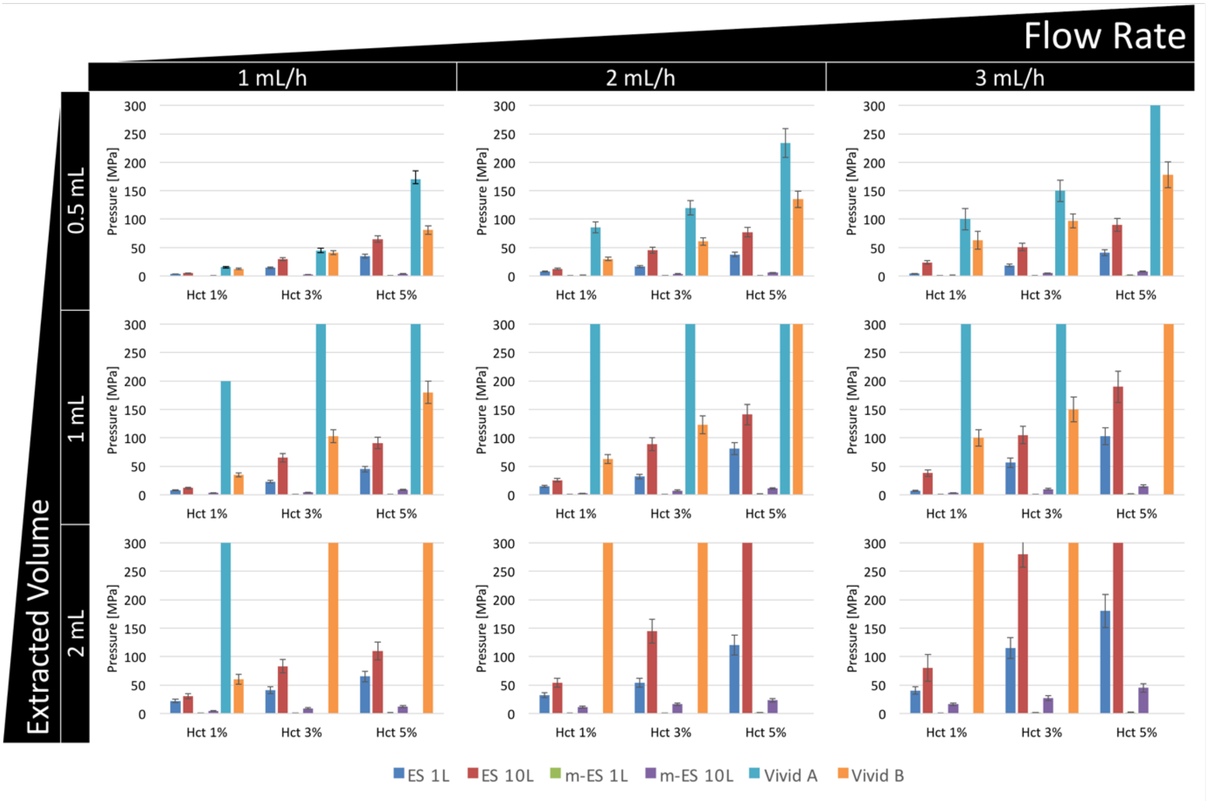

Figure S2: Maximum pressure measured in the MFU as a function of the extracted plasma volume, inlet blood flow rate, Hct_{in} and the kind of membrane.
